# *Chlamydia trachomatis* and human herpesvirus 6 infections in ovarian cancer—Casual or causal?

**DOI:** 10.1371/journal.ppat.1008055

**Published:** 2019-11-07

**Authors:** Nitish Gulve, Thomas Rudel

**Affiliations:** Department of Microbiology, Biocenter, University of Wuerzburg, Wuerzburg, Germany; University of Utah, UNITED STATES

Ovarian cancer is one of the most lethal gynecological malignancies in the world. In the United States, more than 20,000 cases of ovarian cancer average every year, causing more than 14,000 deaths per year (www.cancer.org). This high percentage of mortality arises predominantly due to the silent nature of the disease. Ovarian cancer is diagnosed mostly in the late stages thus earning the disease its name—"Silent killer.” It is therefore of utmost importance to identify any markers that will allow early detection of ovarian cancer.

The National Cancer Institute associates 15%–20% of all cancer with infectious agents. Studies in the past have shown the presence of several viral and bacterial markers in ovarian cancer samples [1, 2]. Understanding the molecular mechanisms of pathogenesis of these oncogenic pathogens may therefore enable early intervention in treatment and care of ovarian cancer patients. In this brief review, we endeavor to highlight the role that coinfection of human herpesvirus 6 (HHV-6) and *Chlamydia trachomatis* may play in initiation and progression of ovarian cancer and propose a theory that may justify their presence in ovarian cancer tissues, thus enabling a directed therapeutic approach.

*C*. *trachomatis* is an obligate intracellular, gram-negative bacterium that is transmitted sexually. More than 2.8 million cases are registered in the US alone [3]. However, the actual number is believed to be much higher, owing to the asymptomatic nature of most *C*. *trachomatis* infections. *C*. *trachomatis* has a 48–72 hour life cycle in which it infects the cells, replicates, and exits by host cell lysis. During its developmental cycle, *C*. *trachomatis* cycles between 2 forms—infectious elementary bodies and replicative reticulate bodies (RB). Its presence in the cell is confined to a vacuole- inclusion. One characteristic of *C*. *trachomatis* infection is its ability to persist in an individual for months up to years. It modulates the host-cell signaling pathways, interacts with various organelles, and evades apoptosis to enable the completion of its developmental cycle [4]. In its pursuit of survival, however, *C*. *trachomatis* infection induces reactive oxygen species (ROS) production via the NADPH and NOD-like receptor family member X1 (NLRX1) pathways [5]. ROS further leads to oxidative damage of DNA, which is further repaired by base excision repair (BER) and nucleotide excision repair (NER) pathways. Recent studies have shown that *C*. *trachomatis* impairs BER of damaged DNA by down-regulating polymerase beta [6]. Deficiency in BER pathway enables the cells to acquire tumorigenic properties [7]. Inefficient BER leads to accumulation of single strand breaks which eventually lead to double strand breaks in the DNA [8]. Telomeres, the protective molecular caps on chromosomes, are damaged through induced telomere shortening during *C*. *trachomatis* infection [9]. *C*. *trachomatis* also affects the DNA damage response and associated signaling of DNA double strand break and telomere repair [10–12]. During *C*. *trachomatis* infection, the host cell encounters DNA damage and suffers impaired repair thereby giving rise to the underlying foundation of the prominent cancer hallmark—genomic instability.

HHV-6 is a betaherpesvirus that has a double-stranded DNA genome. It infects nearly every individual by the age of 2 years. Its unique ability of integration in host telomeres enables it to maintain a lifelong latency in the infected individual. It mediates this integration through homologous recombination between its direct repeat (DR) sequences and host telomeric sequences. This integrated state is termed as chromosomally integrated HHV-6 (ciHHV-6) [13]. This integrated virus can be transmitted vertically in a mendelian fashion and is then termed as inherited chromosomally integrated HHV-6 (iciHHV-6). iciHHV-6 occurs in 1% of the general population in which at least 1 copy of the virus is present in every nucleated cell of the body [14]. This integrated virus may reactivate further in the lifetime of an individual by telomere-circle formation mechanism, which causes the excision of virus and its replication and/or transcription [9]. HHV-6 reactivation can occur due to many reasons, predominantly by stress and immunosuppression. Reactivation of HHV-6 is associated with a wide range of disorders [15–17]. Interestingly, DR sequences are able to integrate within the host genome even in absence of the viral genome. Both in vivo and in vitro studies have shown that viral DRs are capable of integrating in telomeric, as well as in nontelomeric, regions of host chromosomes [18]. Here, the viral DRs were shown to integrate in the intronic regions of gene encoding angiogenesis factor AGGF1 and G alpha interacting protein GAIP [18]. Integration of viral elements in the intronic regions may lead to enhanced gene expression [19]. This transposon-like feature of HHV-6 DR bears the potential of disrupting the regulation of important genes of human genome. The randomness of DR integration makes it an even more lethal cause of genomic instability. Recently, early reactivation or transactivation of HHV-6 has been highlighted by identifying small noncoding viral RNAs (sncRNAs) and their effect on the host transcriptome [20]. The viral DR encoded DR7 protein is known to bind tumor suppressor p53 and inhibit its nuclear translocation by sequestering it in cytoplasm. This strategy of HHV-6 to evade apoptosis may suffice as an initial trigger towards tumorigenesis [21].

**Fig 1:**
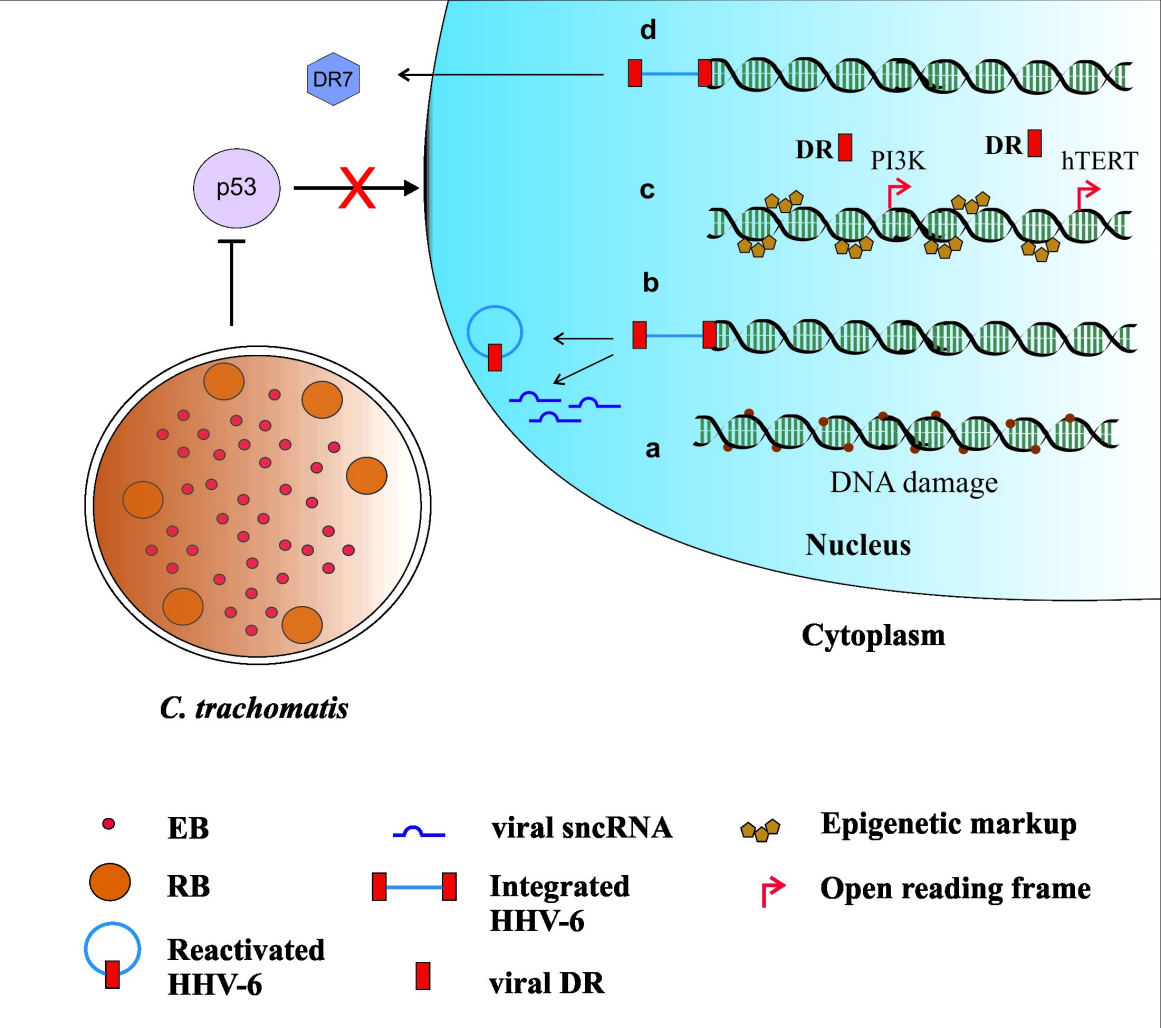
Consequences of *C*. *trachomatis* and HHV-6 coinfection. *C*. *trachomatis* infection of an iciHHV-6 cell leads to (a) DNA damage due to impaired BER and other pathways of DNA damage signaling and (b) HHV-6 reactivation or transactivation and may also lead to transcription of viral sncRNAs. *C*. *trachomatis* changes the epigenetic markup of host cells causing global heterochromatin formation (c). HHV-6 reactivation or transactivation on the other hand may cause (c) integration of DR sequences at regions in host genome that are “active” during *C*. *trachomatis* infection. Integration at important open reading frames of important genes such as PI3K or hTERT may promote transformation of the cell. HHV-6 DR encodes an oncoprotein DR7, which binds and sequesters p53 in the cytoplasm (d). p53 is also down-regulated in *C*. *trachomatis* -infected cells by various mechanisms. BER, base excision repair; HHV-6, human herpesvirus 6; iciHHV-6; inherited chromosomally integrated HHV-6; sncRNAs, small noncoding viral RNAs.

*C*. *trachomatis* and HHV-6 share an interesting dynamic of coinfection. Coinfection of a *C*. *trachomatis* infected cell with HHV-6 induces *C*. *trachomatis* persistence in vitro [22], whereas *C*. *trachomatis* infection of a latent HHV-6 cell line induces reactivation of the virus [9]. Both these scenarios are detrimental to the genome stability of the host cell. Persistence of *C*. *trachomatis* would mean DNA damage over an extended period of time, whereas reactivation of virus may induce production of viral sncRNAs, and random DR integration may severally hamper genome stability (Fig 1). *C*. *trachomatis*, although being associated with ovarian cancer for nearly a decade now, is mostly studied in its active infectious state. The persistence model of *C*. *trachomatis* is seldom focused upon by researchers. Time and again, epidemiological studies employing extensive controls have pinpointed past *C*. *trachomatis* infections to ovarian cancer [23]. A recent study using PathoChip array was employed to identify various pathogenic signatures in ovarian cancer samples. The hybridization signal to pathogen genomic material was compared with both matched and unmatched control samples. Astonishingly, high HHV-6 signals were detected in ovarian cancer but not in either of the control samples. *Chlamydia* was present with a low prevalence in the same study [1].

Could these pathogens act synergistically and bring about transformation in ovarian cells? Several studies have reported that pathogens do co-occur and coinfect, and such coinfections are implicated in different types of cancer. *C*. *trachomatis* has been known to be an important factor in determining the course of Human Papillomavirus (HPV) infection and *C*. *trachomatis*/HPV coinfection may cause cervical cancer [24–26]. *Plasmodium falciparum* and Epstein Barr virus (EBV) coinfection is implicated in Burkitt Lymphoma in children in equatorial Africa [27]. *Helicobacter pylori* and Hepatitis C virus (HCV) are often implicated as coinfecting pathogens in a range of abnormalities, including liver cirrhosis, non-Hodgkin’s lymphoma, and gastric adenocarcinoma [28–30]. However, currently there is no study focusing on HHV-6 and *C*. *trachomatis* coinfections in cancer samples. It is probably time to strip HHV-6 off its “benign” label and consider its coinfection with *C*. *trachomatis* and/or other pathogens for further in-depth studies. Identification of prevalence rates of coinfection in ovarian cancer samples may enable researchers to step-up the in vitro studies and move towards more robust models to study molecular pathogenesis of coinfection. *C*. *trachomatis* down-regulates p53 by various mechanisms to evade apoptosis [31, 32]. Hence, therapies directed towards stabilizing p53 during infection could be further explored to reduce *C*. *trachomatis*-induced onset of ovarian cancer. *C*. *trachomatis* also changes the miRNA profile of the host cell such as by up-regulating miR-30c or miR-499a targeting DRP-1 and polymerase beta, respectively [6, 33]. Both miRNAs also target p53. Therefore, research on miRNA inhibitors as a preventive measure during infection could be considered as another approach. Strong correlation of past infection with *C*. *trachomatis* with nearly absent or low prevalence of pathogen in the cancer tissue suggests the ability of this pathogen to alter cells in such a way that further escalates and leads to transformation even after the pathogen is cleared. Down-regulation of p53 and induction of DNA damage are characteristics of *C*. *trachomatis* infection that would fit almost perfectly with this hypothesis. However, preexisting genomic malady such as iciHHV-6 could further enhance the magnitude of *C*. *trachomatis-*induced genomic instability and mediated oncogenesis. *C*. *trachomatis* causes global heterochromatin formation of host genome [10]. Therefore, when most of the genome is inaccessible, HHV-6 reactivation during *C*. *trachomatis* infection may lead to DR integration at chromosomal regions that are “active” or accessible. Genes, which are up-regulated during *C*. *trachomatis* infection, therefore, form tangible targets for DR integration. Genetic counseling for iciHHV-6 status owing to the hazardous nature of DR integration should therefore be considered for predisposed individuals. One additional marker enabling early detection of ovarian cancer will go a long way in reducing the burden of the disease and allowing a directed therapeutic approach.

Decades have passed after the Hippocratic dyad explaining that health is achieved by man-environment harmony, and that dyad has since been upgraded to a triad to include the etiological agent. Although many infectious agents causing cancer such as HPV, EBV, or *Helicobacter pylori* have been well-studied in terms of their molecular mechanism causing cancer, others like *C*. *trachomatis* and HHV-6, albeit strongly, are merely associated with cancer. It is perhaps time to design more comprehensive studies and harness “omics” approaches to understand the possibility of these coinfections in ovarian cancer and subsequently identify the molecular mechanisms.
